# Risk of acute gastroenteritis associated with human bocavirus infection in children: A systematic review and meta-analysis

**DOI:** 10.1371/journal.pone.0184833

**Published:** 2017-09-14

**Authors:** Ri De, Liying Liu, Yuan Qian, Runan Zhu, Jie Deng, Fang Wang, Yu Sun, Huijin Dong, Liping Jia, Linqing Zhao

**Affiliations:** Laboratory of Virology, Beijing Key Laboratory of Etiology of Viral Diseases in Children, Capital Institute of Pediatrics, Beijing, China; University of Kansas Medical Center, UNITED STATES

## Abstract

Human bocaviruses (HBoVs), which were first identified in 2005 and are composed of genotypes 1–4, have been increasingly detected worldwide in pediatric patients with acute gastroenteritis. To investigate if HBoV infection is a risk factor of acute gastroenteritis in children younger than 5 years old, we searched PubMed, Embase (via Ovid), the Chinese Biomedical Literature Database (CBM), and the Cochrane Library for studies assessing the prevalence of HBoVs in individuals from Oct 25, 2005 to Oct 31, 2016. We included studies using PCR-based diagnostics for HBoVs from stool specimens of patients with or without acute gastroenteritis that carried out research for over 1 year on pediatric patients aged younger than 5 years old. The primary outcome was the HBoV prevalence among all cases with acute gastroenteritis. Pooled estimates of the HBoV prevalence were then generated by fitting linear mixed effect meta-regression models. Of the 36 studies included, the pooled HBoV prevalence in 20,591 patients with acute gastroenteritis was 6.90% (95% confidence interval (95% CI): 5.80–8.10%). In the ten studies with a control group, HBoVs were detected in 12.40% of the 3,620 cases with acute gastroenteritis and in 12.22% of the 2,030 control children (odds ratio (OR): 1.44; 95% CI: 0.95–2.19, *p* = 0.09 between case and control groups). HBoV1 and HBoV2 were detected in 3.49% and 8.59% of acute gastroenteritis cases, respectively, and in 2.22% and 5.09% of control children, respectively (OR: 1.40; 95% CI: 0.61–3.25; *p* = 0.43 and OR: 1.68; 95% CI: 1.21–2.32; *p* = 0.002, respectively). Current evidence suggests that the overall HBoV prevalence in children younger than 5 years old is not significantly different between groups with or without acute gastroenteritis. However, when HBoV1 was excluded, the HBoV2 prevalence was significantly different between these two groups, which may imply that HBoV2 is a risk factor of acute gastroenteritis in children younger than 5 years old.

## Introduction

Acute gastroenteritis is one of the most common pediatric diseases. Its morbidity remains high among infants and young children, and acute gastroenteritis causes an estimated three million deaths annually as well as severe clinical problems and a high social burden. Although acute gastroenteritis is a common disease and a major public health problem worldwide, the etiological agent is not diagnosed in nearly 40% of cases [1–4].

On Oct 25, 2005, Allander et al. found that a novel virus, human bocavirus (family Parvoviridae, subfamily Parvovirinae, genus *Bocaparvovirus*), then named HBoV1, was associated with respiratory tract infection in children [5, 6]. In 2009–2010, three additional HBoV genotypes (HBoV2–4) were reported [7–13], inspiring interest in the role of HBoVs in children. A growing number of studies have subsequently shown that HBoV is frequently detected in the feces of children with acute gastroenteritis [14–16]. Some case-control studies reported that HBoV is just a bystander in acute gastroenteritis, but that finding disagrees with the results of other studies on this topic [17]. Given the conflicting reports, the field has not yet reached a consensus about the role of HBoVs in acute gastroenteritis. Thus, additional work is needed to determine if HBoVs have a pathogenic role in acute gastroenteritis. Here, we performed a systematic review and meta-analysis to investigate the role of HBoVs in pediatric patients younger than 5 years old who have acute gastroenteritis.

## Materials and methods

### Search strategy

**Fig 1:**
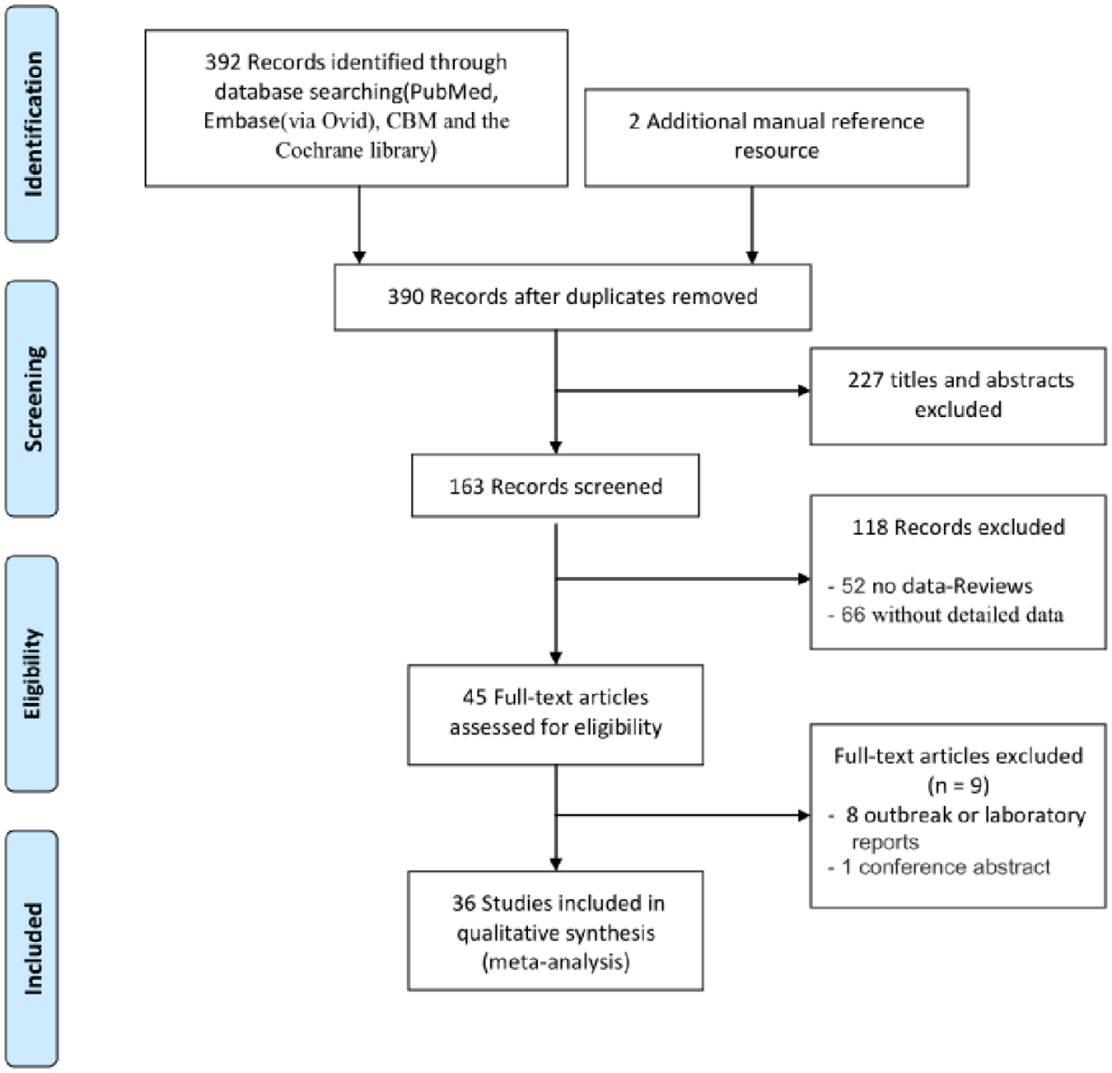
Flow chart of the literature search. The screening, and assessing of HBoV infection data for eligibility, and selecting articles for the meta-analysis.

Following the Preferred Reporting Items for Systematic Reviews and Meta-Analyses (PRISMA) recommendations [18], we performed a systematic, internet-based search using Pubmed, Embase (via Ovid), CBM(Chinese bibliographic database of biomedicine), and the Cochrane Library. The Boolean logic method was used to search all full articles. Fig 1 shows a list of the index or primary keywords used to search English and non-English studies. Since the initial evidence of HBoV infections was reported on Oct 25, 2005 by Allander et al. [5], our temporal limit for the search was set from Oct 25, 2005 to the date on which our study began, Oct 31, 2016.

### Inclusion and exclusion criteria

After receiving training, two researchers independently reviewed the abstracts identified by keyword searches and selected articles for further detailed assessment according to the inclusion and exclusion criteria. When a study met the three requisite terms, 1) used PCR-based diagnostics for HBoV in stool specimens from patients with acute gastroenteritis infection or part of an asymptomatic control group, 2) carried out their research over at least 1 year in a specified area, and 3) studied pediatric patients aged under 5 years old, we downloaded the full manuscript text to organize or compress the qualified article in the next step. Studies were excluded if they were duplicate documents, had a lack of particular data, such as the number of HBoV-positive patients with acute gastroenteritis, had a lack of comparability between the case group and control group because they were collected from different hospitals or communities, or if they had low quality assessment scores.

### Data extraction and stratification

The following information was extracted from each study when it was available: first author, year of publication, country, diagnostic method used, number of cases tested, number or rate of HBoV-positive cases, number of controls tested, and number or rate of positive controls. Data were stratified for descriptive analyses by the study type (with or without a control group), continent, diagnostic method, and HBoV prevalence level.

### Statistical analysis

We generated pooled estimates of HBoV prevalence as the outcome in all pediatric patients with acute gastroenteritis who were under 5 years of age by using the software Stata 12.0. We used an unrestricted maximum likelihood linear random-effect meta-regression analysis that included global distribution (Asia, America, Europe, and Australia) and diagnostic methods (classical PCR, nested-PCR or semi-nested PCR, and real time PCR) as regressors for residual heterogeneity. The significant differences of the pooled HBoV prevalence were determined by using one-way ANOVA test of SPSS 20.0 (*p* < 0.05).

For studies with a control group, two of the authors of this manuscript assessed the quality of each by using the Newcastle-Ottawa Scale (NOS) in the three domains that are recommended by the Cochrane Collaboration [19]: the selection of study groups, the comparability of groups, and the exposure of groups.

Studies that received one star in every domain were judged as being of high quality. The data were then extracted from each study to calculate odds ratios (ORs) for risk effect estimates by a random effects model (DerSimonian and Laird) [20]. As a weighted average, an OR value that was located in the confidence region (*p* < 0.05) and greater than 1 suggested a higher risk factor. Cochrane’s Q statistic and the I^2^ index statistic, according to the method suggested by Higgins [21], were calculated for each analysis as a measure of the proportion of the overall variation that was attributable to between-study heterogeneity. An I^2^ of <25% indicates a low degree of heterogeneity, and an I^2^ of ≥75% suggests a high degree of heterogeneity. Egger’s test was used to evaluate the publication bias of studies with a control group, and a *p*-value of <0.05 was taken to indicate a significant publication bias. We performed several sensitivity analysis to test the robustness of our findings. All case-control studies were assessed with the software Stata 12.0.

## Results

### Search results and study selection

Based on the search strategy, we identified 392 studies from PubMed, Embase, CBM, and the Cochrane Library and 2 additional studies from the references of the identified studies. Of these, 4 duplicates, 227 with titles and abstracts only, 52 reviews, 66 without detailed data, 8 about outbreak or laboratory weekly reports, and 1 conference abstract were excluded (Fig 1 and S1 PRISMA Checklist). This left a final data base consists of 36 studies, including 10 studies with a control group.

### Data extraction

We extracted data from the included 36 original publications and used it to form an Excel database (Table 1). The included studies were all published between Oct 25, 2005 and Oct 31, 2016, and they included data from 18 countries (Brazil, United States, Germany, Russia, Spain, Hungary, UK, Milan, Finland, China, Iranian, India, Pakistani, Japan, Korea, Thailand, Albania, and Australia). Overall, the systematic review included 20,591 children who were younger than 5 years of age. The studies were divided into four groups according to the continents on which the research had been performed (21 from Asia, 4 from America, 10 from Europe, and 1 from Australia) as well as into three groups based on the PCR method that had been used by the study (classical PCR group: 18 studies; real-time PCR group: 11 studies; and nested/semi-nested PCR group: 7 studies). Ten of these 36 studies contained control group data and met the criteria for NOS; these were applied to a meta-analysis composed of 5,650 cases (3,620 acute gastroenteritis patients and 2,030 healthy controls). The HBoV prevalence results of these studies were divided into different levels, ranging from <1% to >10%.

**Table 1:** Distribution of data among various strata from the 36 studies selected for meta-analysis.

	Number of studies (N = 36)	Median number of children tested per study (range)	Number of children tested (N = 20591)
Types of studies			
Studies with control-group	10	276(96–878)	3620
Studies without control-group	26	307(47–5250)	16971
Continents			
Europe	10	290(53–5250)	10601
America	4	128(105–782)	1565
Asia	21	365(47–1435)	8239
Australia	1	186(-)	186
Diagnostic method			
Classical PCR	18	354(186–5250)	12951
Nested-/semi-nested PCR	7	110(47–962)	1946
Real-time PCR	11	381(200–1216)	5694
Level of HBoV prevalence (%)			
<1	2	593(225–962)	1187
1–2.5	6	1124(329–5250)	10045
2.5–5	8	263(61–397)	1970
5–7.5	3	418(273–1216)	1907
7.5–10	10	200(45–527)	3113
>10	7	365(47–632)	2369

### Description analysis for the prevalence of HBoVs

**Fig 2:**
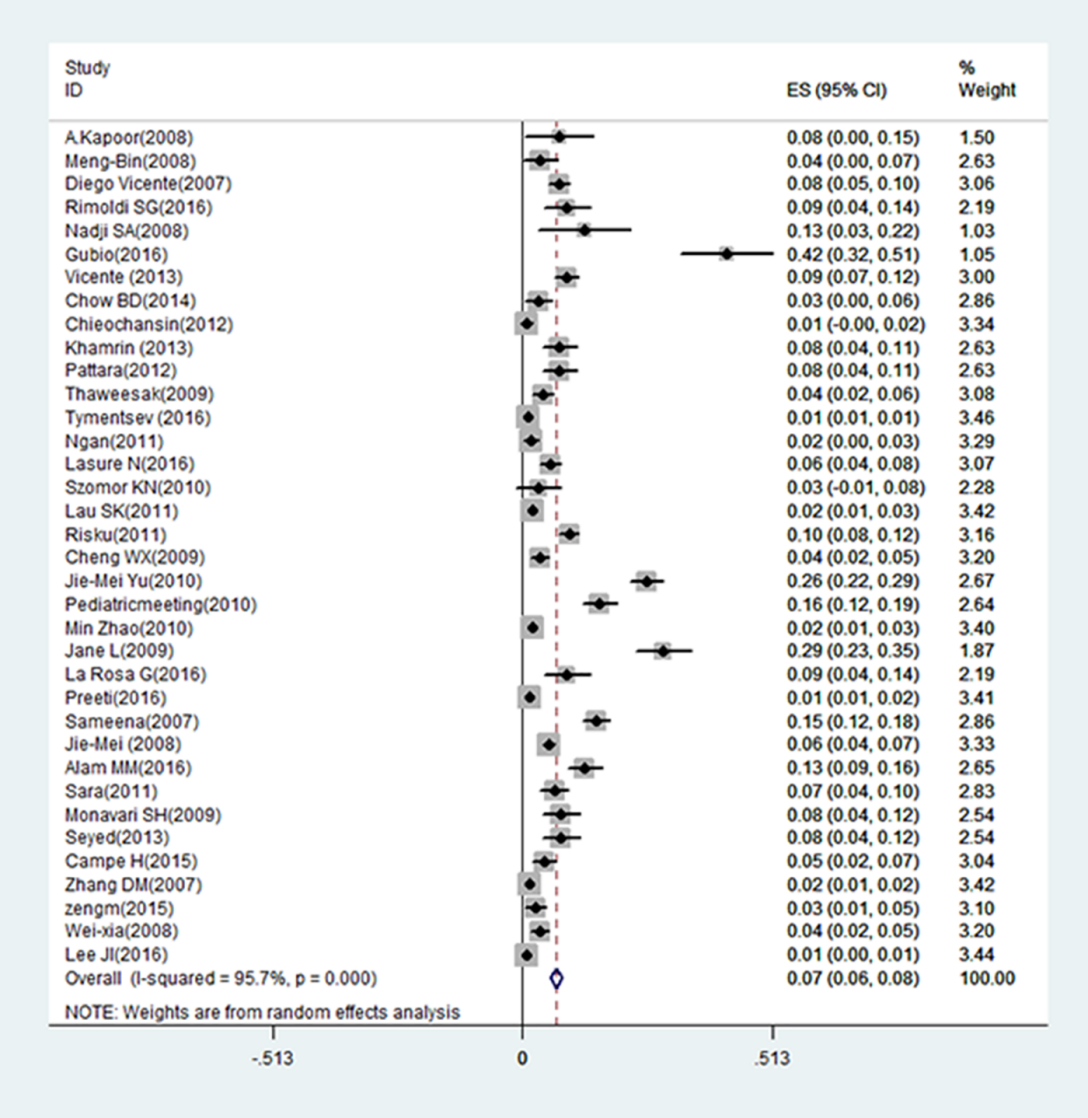
Forest plot showing pooled HBoV prevalence in patients <5 years old with acute gastroenteritis from 36 studies. The ES (Expected shortfall) of pooled prevalence rate of HBoVs is 0.067 (95% CI: 0.056–0.078) using random effects modeled (D+L pool); I-squared (variation in ES attributable to heterogeneity) = 95.4%; estimate of between-study variance Tau^2^ = 0.0009.

Among the included 36 studies, the overall HBoV prevalence in cases with acute gastroenteritis was 6.90% (95% confidence interval (95% CI): 5.80–8.10%) (Fig 2, S1 Text).

**Fig 3:**
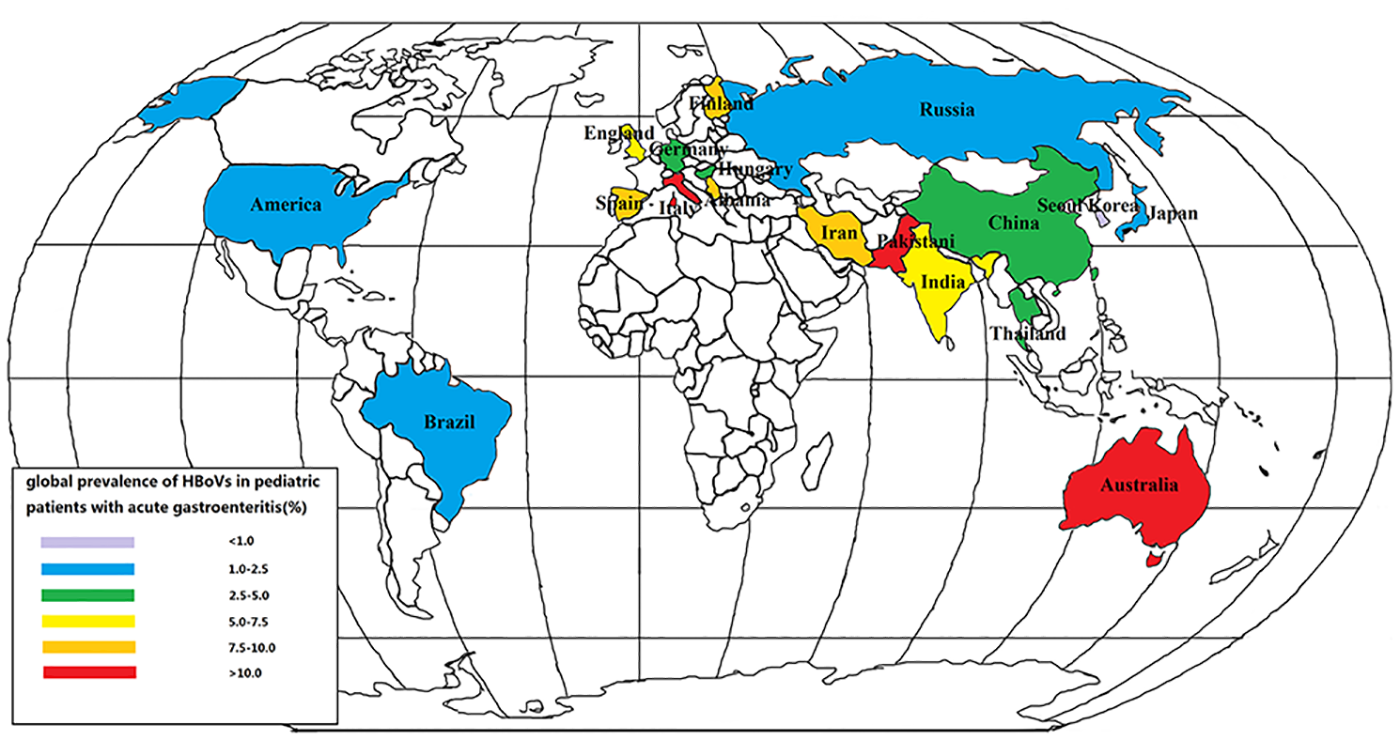
Reported global prevalence of HBoVs in pediatric patients <5 years old with acute gastroenteritis, from 2005 to 2016. Red background indicates countries with >10% HBoVs population; orange background, 7.6–10%; yellow background, 5.1–7.5%; green background, 2.6–5%; blue background, 1.1–2.5%; and purple background, <1% population.

We mapped the global distribution of the HBoV incidences in pediatric patients younger than 5 years old with acute gastroenteritis that were reported from 18 countries during 2005 to 2016. The highest HBoV infection rate was reported in Australia (Fig 3). In China, the HBoV infection prevalence was 2.6–5%, as highlighted by the area shown with a green background in Fig 3, and most countries had 1.1–2.5% of their acute gastroenteritis population reported as positive for HBoV infection, as highlighted by the area with a blue background in Fig 3.

Based on data from 18 countries that were divided into four groups, the prevalence of HBoVs in children younger than 5 years old with acute gastroenteritis was 5.47% (95% CI: 5.04–5.90%; *p* < 0.05) in Asia, 6.90% (95% CI: 6.38–7.45%; *p* < 0.05) in America, 4.07% (95% CI: 2.82–5.33%; *p* < 0.05) in Europe, and 29.03% (only one report) in Australia. The distributions of HBoV prevalence among Asia, America, and Europe showed no statistical significant difference as determined by the ANOVA test.

We analyzed three groups based on the diagnostic methods used by the study. The HBoV prevalence was 4.96% (95% CI: 4.58–5.34%; *p* < 0.05) when assessed by classical PCR, 5.58% (95% CI: 4.98–6.18%; *p* < 0.05) when assessed by real-time PCR, and 6.10% (95% CI: 5.69–6.51%; *p* < 0.05) when assessed by nested- or semi-nested PCR. Although there was no significant difference in HBoV prevalence among these different diagnostic methods, as determined by the ANOVA test, this suggests there is a trend of HBoV prevalence being higher when nested or semi-nested PCR was used compared with when real-time PCR or classical PCR was used.

A meta-regression found that the test of heterogeneity I^2^ = 95.4%, Tau^2^ (Estimate of between-study variance) = 0.0009, adjusted R^2^ (Proportion of between-study variance explained) = 0.75% by variable of areas, and the adjusted R^2^ = 1.91% by variable of methods. These results suggest that the continents on which the study was conducted and the PCR methods used by the studies were both confounders and may be responsible for the detected heterogeneity between studies.

### Meta-analysis for studies with a control group

The 10 of the 36 included studies that had control group data and met the criteria for NOS were applied to a meta-analysis. We performed a quality assessment for each of the individual studies based on the NOS recommended by the Cochrane Collaboration. A study can be awarded a maximum of one star for each numbered item within the selection and exposure categories. A maximum of two stars can be given for Comparability. Studies scoring 7–9, 4–6, and 0–3 are regarded as high-, moderate-, and low-quality, respectively. Of these ten studies, most were assigned over six stars in all three domains, which confirmed that they were of high quality (S2 Text).

**Fig 4:**
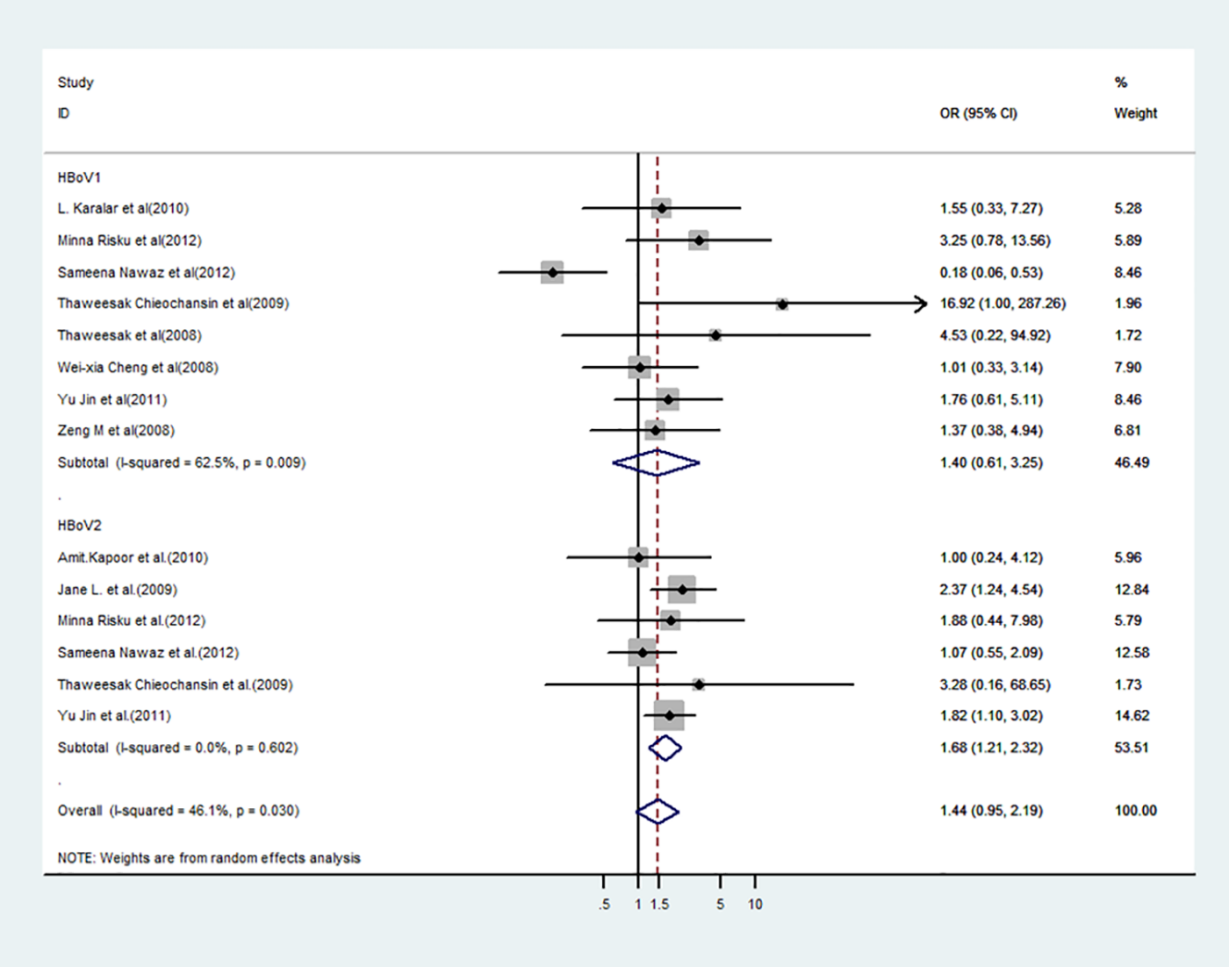
Forest plot of analysis on odds radio according to HBoVs infection and acute gastroenteritis. CI: confidence interval, M–H: Mantel–Haenszel.

Among the 10 studies with a control group that were pooled in a meta-analysis (Table 2), HBoVs were detected in 12.40% (95% CI: 4.65–18.15%; *p* < 0.05) of cases with acute gastroenteritis and in 12.22% (95% CI: 4.39–17.01%; *p* < 0.05) of control children with an OR value of 1.44 (95% CI: 0.95–2.19 and the test of heterogeneity Tau^2^ = 0.25; I^2^ = 46%; *p* = 0.09) between the case and control groups, which revealed no statistical significance (Fig 4).

**Table 2:** HBoV prevalence in different groups among the 10 pooled studies with a control group.

First author (year of publication)	City (Country)	Sample size(n)	In case-group with acute gastroenteritis				In control-group			
			Number of cases (n)	Number of HBoV positive (%)	Number of HBoV1 positive (%)	Number of HBoV2 positive (%)	Number of controls (n)	Number of HBoV positive (%)	Number of HBoV1 positive (%)	Number of HBoV2 positive (%)
Amit.Kapoor (2010)	San Francisco (USA)	192	96	32	2	25	96	24	1	23
Jane L (2009)	Adelaide (Australia)	372	186	54	17	32	186	30	11-	15
L. Karalar (2010)	Regensburg (Germany)	113	53	4	4	-	60	3	3	-
Minna Risku (2012)	Tampere (Finland)	990	878	85	49	29	112	6	1	0
Sameena Nawaz (2012)	Liverpool (United Kingdom)	1290	606	92	4	17	684	153	24	18
Thaweesak Chieochansin (2009)	Bangkok (Tailand)	540	327	14	12	2	213	0	0	0
Thaweesak (2008)	Bangkok (Tailand)	427	225	2	2	-	202	0	0	-
Wei-xia Cheng (2008)	Lan zhou (China)	512	397	14	14	-	115	4	4	-
Yu Jin (2011)	Beijing (China)	794	632	162	27	129	162	24	4	20
Zeng M (2010)	Shanghai (China)	420	220	6	6	-	200	4	4	-
Total		5650	3620	465(12.40)	120(3.49)	234(8.59)	2030	248(12.22)	41(2.22)	76(5.09)

HBoV1 was detected in 3.49% (95% CI: 1.77–5.79%; *p* < 0.05) of cases with acute gastroenteritis and in 2.22% (95% CI: 1.32–3.80%; *p* < 0.05) of control children with an OR value of 1.40 (95% CI: 0.61–3.25) and a *p*-value of >0.05 as determined by the test of heterogeneity (Tau^2^ = 0.84; I^2^ = 63%; *p* = 0.43), which revealed no statistical significant difference. Additionally, the results of an Egger’s test did not suggest the presence of a publication bias (*p* = 0.464).

HBoV2 was detected in 8.59% (95% CI: 2.88–10.60%; *p* < 0.05) of cases with acute gastroenteritis and in 5.09% (95% CI: 1.76–8.72%; *p* < 0.05) of control group children with an OR value of 1.68 (95% CI: 1.21–2.32) and a *p*-value of <0.05 as determined by the test of heterogeneity (Tau^2^ = 0.00; I^2^ = 0%; *p* = 0.002), which revealed a statistical significant difference in the HBoV2-positive rates between the case and control groups. The results of an Egger’s test did not suggest the presence of a publication bias (*p* = 0.464).

We performed a sensitivity analysis to evaluate the effect of all included studies on the pooled ORs by omitting each HBoV group shown in Fig 4 in turn. The pooled ORs were not affected significantly by excluding any study, which revealed that all meta-analytic conclusions remained robust to this testing.

## Discussion

Since HBoV1 was found in 2005, scientists worldwide have spent the last decade investigating the etiological role of HBoV1–4. HBoV1 is predominantly found in the respiratory tract, whereas HBoV2, HBoV3, and HBoV4 are mainly detected in stool [22]. However, many questions concerning HBoV still remain, especially regarding the role of HBoVs in acute gastroenteritis. In this study, we performed a systematic review and meta-analysis to assess if HBoV infection is a risk factor of acute gastroenteritis in children younger than 5 years of age.

Our results provide an updated estimate that HBoVs are associated with 6.90% (95% CI: 5.80–8.10%; *p* < 0.05) of all cases with acute gastroenteritis in children younger than 5 years of age. This finding supports previous descriptions of the human fecal virome in which *Parvoviridae* are frequently detected [17].

Most of the countries included in the present study had 1.1–2.5% of acute gastroenteritis patients reported as positive for HBoV. There is also a trend of decreasing prevalence from Australia (about 29.03%, based on only one study) to America (about 6.90%) to Asia (about 5.47%) to Europe (about 4.07%), as well as from nested- or semi-nested PCR (6.10%) to real-time PCR (5.58%) to PCR (4.96%) groups, but these differences were not statistically significant. The results of our meta-regression analysis show that the continents on which the studies were conducted and the PCR methods used by the studies were both confounders and may be responsible for the heterogeneity between studies. Therefore, it is possible that the differences of HBoV frequencies found among these countries may be related to the methodology used to detect the presence of HBoVs. This conclusion, which is in agreement with a report by Campos et al. [10], supports the importance of conducting an active HBoV surveillance over all continents in the world by using a uniform method.

Pooled estimates of HBoV prevalence were generated by fitting linear mixed effect meta-regression models. In the 10 studies with control groups (Table 2), HBoVs were detected in 12.40% of the 3,620 cases with acute gastroenteritis and in 12.22% of the 2,030 control children with an OR value of 1.44 (95% CI: 0.95–2.19; *p* = 0.09) between the case and control groups, which revealed that there is no statistical significant difference between these two groups (Fig 4). Despite the demonstrated low tropism of HBoVs for the human body, HBoVs were reported to show a high persistence in some sites, particularly in the lymphatic tissue [23], which may explain the high prevalence of HBoVs in the control group. However, when HBoV1, which was detected in 3.49% of acute gastroenteritis cases and in 2.22% of control children with an OR value of 1.40 (95% CI: 0.61–3.25; *p* = 0.43), was excluded, the prevalence of HBoV2, which was detected in 8.59% of acute gastroenteritis cases and in 5.09% of control children with an OR value of 1.68 (95% CI: 1.21–2.32; *p* = 0.002), was significantly different between these two groups of children. The results may imply that HBoV2 is a risk factor for acute gastroenteritis in children younger than 5 years of age, while HBoV1 detected in stools may be the product of viral discharge from the human body. In a review [17], Ong et al. reported the conclusion that HBoV2 was just a bystander in acute gastroenteritis, which is inconsistent with the findings in our work. These opposing conclusions may be due to the different inclusion and exclusion criteria and the selection of different studies in these analyses.

This systematic review has some shortcomings. First, age grouping was highly variable in published reports, which made it difficult to subgroup study populations into finite age groups. Second, not every included study had investigated all four HBoV genotypes. Due to the lower prevalence of some genotypes, it remains to be determined whether or not HBoV3–4 are also associated with acute gastroenteritis. Third, although we tried to reduce the causes of heterogeneity by performing subgroup analysis and regression analysis, the results heterogeneity tests show that the heterogeneity could not be completely eliminated. As such, we have discussed the residual heterogeneity in the clinical diversity and methodological diversity as well as the statistical heterogeneity. Another limitation is the lack of a large number of relevant research data. In particular, the two included case-control studies contain less than 200 samples each, which likely led to selection bias. More data should be accumulated to confirm the conclusion of this study.

## Conclusion

In conclusion, current evidence suggests that even though HBoVs may be considered as a bystander of acute gastroenteritis, HBoV2 infection should be thought as a risk factor of acute gastroenteritis in children under 5 years old when HBoV1 has been excluded. However, this conclusion, which is based on the data provided by molecular methodology, should be treated with caution and should be confirmed with serology research.

## Supporting information

S1 PRISMA Checklist (DOC)

S1 Text Pooled HBoV prevalence of pediatric patients with acute gastroenteritis from 36 studies. (TXT)

S2 Text Methodology quality evaluated according to Newcastle–Ottawa scale recommended by the Cochrane Collaboration. (XLSX)
